# Assessment of a diffusion phantom for quality assurance in brain microstructure diffusion MRI studies

**DOI:** 10.1038/s41598-025-12777-y

**Published:** 2025-07-30

**Authors:** Mattia Ricchi, Aaron Axford, Jordan McGing, Ayaka Shinozaki, Kylie Yeung, Rebecca Mills, Fulvio Zaccagna, Damian J. Tyler, Claudia Testa, James T. Grist

**Affiliations:** 1 Department of Computer Sciences, University of Pisa, Pisa, Italy; 2 National Institute of Nuclear Physics (INFN), Bologna Division, Bologna, Italy; 3 Oxford Centre for Clinical Magnetic Resonance Research, University of Oxford, Oxford, UK; 4 Department of Physiology, Anatomy, and Genetics, University of Oxford, Oxford, UK; 5 Department of Oncology, University of Oxford, Oxford, UK; 6 Department of Radiology, Cambridge University Hospitals, Cambridge, UK; 7 Nuffield Department of Medicine, University of Oxford, Oxford, UK; 8 Department of Physics and Astronomy, University of Bologna, Bologna, Italy; 9 Department of Radiology, Oxford University Hospitals, Oxford, UK

**Keywords:** Phantom, Validation, Diffusion modelling, DWI/DTI/DKI, NODDI, Applied physics, Neuroscience, Magnetic resonance imaging

## Abstract

Diffusion-weighted imaging (DWI) is a key contrast mechanism in MRI which allows for the assessment of microstructural properties of brain tissues by measuring the displacement of water molecules. Several diffusion models, including the tensor (DTI), kurtosis (DKI), and neurite orientation dispersion and density imaging (NODDI), are commonly used in both research and clinical practice. However, there is currently no standardized method for validating the stability and repeatability of these models over time. This study evaluates the use of a DTI phantom as a standard reference for diffusion MRI model validation. The phantom, along with four healthy volunteers, was scanned repeatedly on different days to assess repeatability and stability. The acquired data were fitted to the diffusion models, with repeatability assessed in the phantom using the coefficient of variation (CoV), while stability in vivo was assessed using the repeatability coefficient (RC). The phantom was consecutively scanned eight times to investigate the impact of gradient coil heating on measurement consistency. Results showed that the phantom provided a highly reproducible reference, with CoVs below 5% across repeated and consecutive acquisitions, confirming the robustness of the diffusion models. In vivo, the low RCs indicated that the models remained stable over time, despite potential physiological variability. This study highlights the essential role of phantoms in diffusion MRI research, providing a reference framework for model validation. Future research will expand on this work to a multi-center study to assess inter-scanner variability, potentially incorporating the phantom into calibration protocols to standardize diffusion MRI measurements across different MRI systems.

## Introduction

Magnetic resonance imaging (MRI) is fundamental to the diagnosis, evaluation, and monitoring of neurodegenerative diseases^1,2^ due to its exceptional soft tissue contrast and multi-planar capability. MRI has a wide range of contrast mechanisms, including the measurement of signal attenuation caused by water diffusion within cellular structures^3^.

Diffusion-tensor imaging (DTI)^4,5^ is an MRI technique that has become more commonly used as both a scientific and clinical tool^6^. By exploiting the random motion of water molecules, DTI can be used to visualize and analyze white matter tracts where water diffusion aligns with the direction of fibers^7^. The conventional in-clinic approach to DTI focuses on metrics such as fractional anisotropy (FA), a measure of the degree of diffusion anisotropy, and mean diffusivity (MD), which describes the average diffusion of water molecules within brain structures^7^.

One limitation of conventional DTI post-processing is the assumption of Gaussian diffusivity: for time periods around tens of milliseconds, the intricate structure of most tissues, composed of various cell types and membranes, can lead the diffusion displacement probability distribution to significantly deviate from a Gaussian shape^8^. To consider and quantify this deviation from Gaussian behavior, Diffusion Kurtosis imaging (DKI) was introduced^9^. DKI provides additional metrics to the standard DTI model. Mean Kurtosis (MK) estimates the average deviation from Gaussian diffusion across all diffusion directions, while axial Kurtosis (AK) quantifies kurtosis along the principal diffusion direction^10^. A limitation of DKI is its high sensitivity to noise and image artefacts^11–13^. For example, because of low radial diffusivities, standard kurtosis estimates in regions with well-aligned voxels may yield low or negative values. To address this, kurtosis can be characterized by the average signals across all directions acquired for each b-value, a method known as mean signal diffusion kurtosis imaging (MSDKI)^11–13^.

DTI and DKI metrics can be used for examining the condition of tissues at a microscopic level, specifically the white matter tracts in the healthy brain and changes associated with pathology. Changes in FA, MD, MK and AK have been observed in various diseases^14–17^as well as disruption of tracts in the oncological setting^17^. Although these markers are characterized by great sensitivity, they are inherently non-specific^18,19^. Therefore, changes in these statistics cannot be attributed to a specific change in the tissue microstructure as different pathological conditions can cause the same effect.

Zhang et al.^19^ proposed a model to address the limitations of the diffusion models and developed a clinically feasible technique for in vivo neurite orientation dispersion and density imaging (NODDI). This technique combines a three-compartment tissue model with at least two-shell high-angular-resolution diffusion imaging (HARDI) protocol, optimized for clinical feasibility, to map neurite orientation dispersion and density in-vivo^19^. NODDI imaging has been quickly adopted in the field of neuroimaging because it can be used to measure changes in microstructures in grey matter (GM) and white matter (WM) with an efficient imaging processing technique^20–22^. However, one limitation of NODDI is that it cannot accurately model intricate neurite configurations that result from the fanning and bending of axons^20^. Tariq et al.^20^ introduced an extension to the NODDI formalism, using the Bingham distribution to characterize neurite dispersion. The Bingham-NODDI estimates the extent of dispersion about the dominant orientation, separately along the primary and secondary dispersion orientations, enabling the characterization of anisotropic orientation dispersion^20^. A further major drawback is the assumption of a fixed, arbitrary diffusivity value for the intra-neurite compartment^19^. This fixed value does not account for biological variability, e.g. across individuals, brain regions, and can introduce bias in the estimated microstructural parameters, such as the orientation dispersion index (ODI), if the true underlying diffusivity deviates from this assumption.

Although diffusion MRI is widely used in clinical practice, for characterizing brain tissue at a microscopic level, it has certain variables and limitations that need to be considered. There is limited understanding of the various factors that may influence the results of brain structure measurements, including hydration and blood pressure^23^. Additionally, various physiological factors, such as aging and gender, can influence DTI-derived FA and MD values^23^. Furthermore, there is a possibility that small changes in brain volume estimates caused by physiological factors such as dehydration, blood pressure, caffeine levels, and circadian rhythm could confound FA and MD^24^. In addition, there is yet no gold standard for validating diffusion measures, as they may vary depending on factors such as scanner type^25^scanning protocols and methods of the software. Currently, a limited number of studies have evaluated the reliability of DTI metrics using HARDI^26,27^.

While the NODDI model promises to provide more specific information about the microstructure of white matter compared to the diffusion tensor alone, its reproducibility can vary depending on several factors and must be carefully evaluated, particularly in longitudinal research settings and clinical populations^28^. This is despite its great potential in characterizing the brain tissue at microscopic level: changes in the dispersion of the orientation or morphology of the axons and dendrites could indicate the emergence of neurological diseases such as multiple sclerosis^29,30^ or Alzheimer’s disease^22,31,32^.

A few studies can be found in literature examining the consistency and repeatability over time of NODDI metrics, highlighting the importance of the choice of the analysis pipeline, the intensity of the magnetic field $\:{\text{B}}_{0}$, and the characteristics of the studied population, as they can influence the stability and the interpretation of the cerebral measures^33,34^. In particular, the reproducibility of the NODDI model can vary depending on several factors and must be carefully evaluated, especially in cases of longitudinal studies and clinical populations. Chung et al.^33^ showed how the strength of the main magnetic field influences the estimated NODDI and DTI parameters, demonstrating the importance of considering this influence in study design and interpretation of results. Lehmann et al.^34^ evaluated the longitudinal reproducibility of several DWI metrics, including DTI and NODDI, using different analysis pipelines. The authors highlighted the need for robust and reproducible methods for the study of microstructural white matter properties, showing how different pipelines may lead to different levels of reproducibility.

There is a particularly evident lack of a standard reference to assess the optimal analytical approach and to evaluate the stability and consistency of the diffusion metrics over time in an unbiased way^35^. A diffusion phantom could provide such ground truth as its microstructural properties would be known and stable over time. As the reproducibility of diffusion biophysical models like NODDI depends not only on the analytical approach but also on the quality of the underlying data^34^using a phantom would allow researchers to isolate and study the impact of technical factors such as the signal-to-noise ratio (SNR) and imaging system-induced distortions on the stability of NODDI metrics. Finally, physiological variability, including potential differences in hydration, movement, or neurophysiological states, can introduce confounding factors. By contrast, the phantom offers a controlled, repeatable reference that eliminates these sources of variability, establishing a stable foundation for validating diffusion MRI protocols.

In this work we introduce the use of a diffusion phantom^36^ as a reference for the evaluation of the consistency and reproducibility of the most used diffusion magnetic resonance measures, in particular DTI, DKI and NODDI models. The phantom mimics restricted anisotropic diffusion in the brain, particularly in white matter. The phantom was scanned on four different days over 2 months and multiple times during the same day using the same scanner and the same acquisition protocol. After evaluating the repeatability of DTI, DKI and NODDI fit results in the phantom, a cohort of four healthy volunteers were scanned twice on the same day, over a 1-month period, to verify the consistency of results in-vivo.

## Results

To assess the repeatability of measurements, the fiber-ring phantom was scanned four times over different days in a 2-month period with a HARDI-type protocol^19,20^. Furthermore, eight consecutive scans were performed, without stopping between each acquisition, on the same day. For each acquisition, the diffusion metrics were extracted from the fiber ring of the phantom using an operator drawn region of interest (ROI). FA values in agreement with those provided by the manufacturer^37^.

**Fig. 1:**
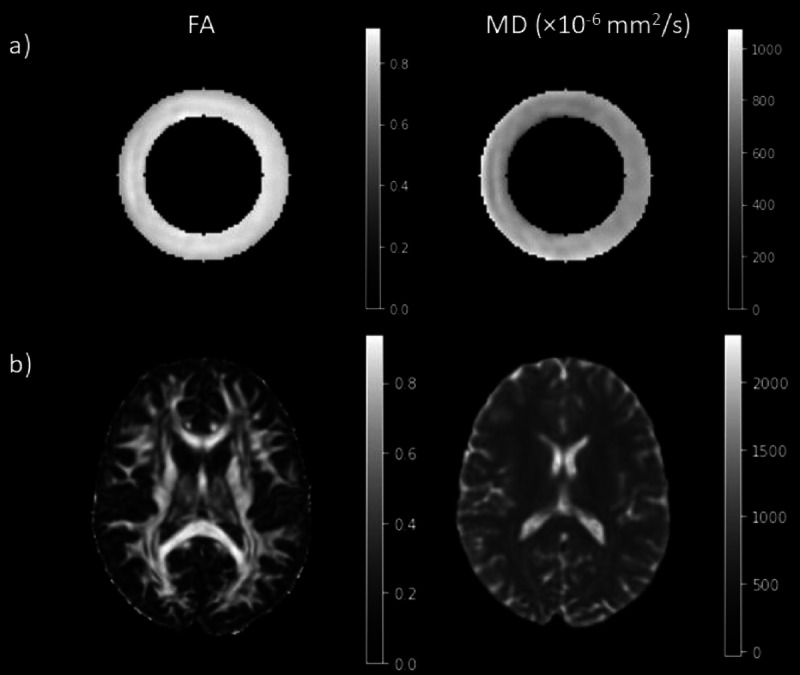
DTI maps obtained in the fiber ring of the phantom (**a**) and in the brain (**b**). The maps show the FA on the left and the MD on the right.

Results for FA and MD values extracted from the inner fiber ring over the four different days are presented in Table 1. The coefficients of variation (CoVs) for the four acquisitions on different days were 1.03% and 2.34% for FA and MD, respectively, both of which were lower than a 5% threshold. This threshold was chosen as having a CoV of 5% would mean that the standard deviation represents 5% of the mean, implying low relative variability. Figure 1 presents the DTI maps showing how the FA and MD values are uniform throughout the fiber ring.

**Table 1:** DTI, DKI and NODDI Bingham-model results for the four scans in different days over a total of 2 months. Metrics were extracted from the fiber ring of the phantom.

	Scan 1	Scan 2	Scan 3	Scan 4	CoV
FA	0.79 ± 0.05	0.8 ± 0.1	0.81 ± 0.04	0.81 ± 0.05	1.03%
MD (×10^− 3^ mm^2^/s)	0.85 ± 0.08	0.8 ± 0.1	0.81 ± 0.08	0.82 ± 0.08	2.34%
DKI AK	0.17 ± 0.13	0.17 ± 0.13	0.19 ± 0.15	0.17 ± 0.13	4.95%
DKI MK	0.56 ± 0.13	0.57 ± 0.13	0.51 ± 0.17	0.56 ± 0.13	4.26%
MSDKI MK	0.83 ± 0.11	0.82 ± 0.11	0.77 ± 0.13	0.81 ± 0.11	2.82%
MSDKI MD (×10^− 3^ mm^2^/s)	0.89 ± 0.12	0.97 ± 0.12	0.97 ± 0.19	0.98 ± 0.14	3.81%
β – fraction	0.36 ± 0.22	0.41 ± 0.25	0.37 ± 0.25	0.42 ± 0.21	6.5%
ODI	0.021 ± 0.002	0.0202 ± 0.0015	0.0202 ± 0.0009	0.0201 ± 0.0007	1.36%
Tissue volume fraction	0.98 ± 0.04	0.96 ± 0.05	0.94 ± 0.09	0.96 ± 0.04	1.47%
Intra – neurite volume fraction	0.45 ± 0.08	0.46 ± 0.08	0.44 ± 0.08	0.45 ± 0.07	1.57%
MSE	0.0011 ± 0.0005	0.0016 ± 0.0016	0.0014 ± 0.0006	0.0012 ± 0.0004	14.5%

To investigate whether repeated data acquisition and the resulting heating of the MRI scanner gradient coils could affect the accuracy of phantom measurements, we conducted a series of eight repeated measurements on the same day. The DTI metrics for the eight consecutive scans demonstrated low CoVs for FA (0.54%) and MD (0.61%), as shown in Table 2.

**Table 2:** DTI, DKI and Bingham-NODDI model results for the eight scans in a row. Metrics were extracted from the fiber ring of the phantom.

	Scan 1	Scan 2	Scan 3	Scan 4	Scan 5	Scan 6	Scan 7	Scan 8	CoV
FA	0.81 ± 0.04	0.80 ± 0.06	0.80 ± 0.05	0.80 ± 0.06	0.80 ± 0.06	0.80 ± 0.06	0.80 ± 0.05	0.81 ± 0.05	0.54%
MD (×10^− 3^ mm^2^/s)	0.81 ± 0.08	0.82 ± 0.07	0.82 ± 0.08	0.82 ± 0.08	0.82 ± 0.07	0.82 ± 0.08	0.83 ± 0.07	0.82 ± 0.07	0.61%
DKI AK	0.19 ± 0.15	0.19 ± 0.14	0.23 ± 0.16	0.19 ± 0.14	0.22 ± 0.16	0.24 ± 0.17	0.19 ± 0.14	0.20 ± 0.15	9.36%
DKI MK	0.51 ± 0.17	0.51 ± 0.13	0.52 ± 0.15	0.51 ± 0.13	0.51 ± 0.13	0.52 ± 0.15	0.51 ± 0.13	0.51 ± 0.13	0.84%
MSDKI MK	0.77 ± 0.13	0.81 ± 0.10	0.79 ± 0.11	0.79 ± 0.11	0.79 ± 0.11	0.80 ± 0.11	0.79 ± 0.10	0.80 ± 0.11	1.37%
MSDKI MD (×10^− 3^ mm^2^/s)	0.97 ± 0.19	0.95 ± 0.12	0.98 ± 0.16	0.97 ± 0.13	0.98 ± 0.14	0.98 ± 0.15	0.97 ± 0.13	0.98 ± 0.14	0.99%
β – fraction	0.37 ± 0.25	0.37 ± 0.24	0.37 ± 0.24	0.36 ± 0.25	0.35 ± 0.24	0.38 ± 0.24	0.36 ± 0.24	0.37 ± 0.23	2.34%
ODI	0.0202 ± 0.0009	0.0202 ± 0.0009	0.0202 ± 0.0009	0.0202 ± 0.0009	0.0202 ± 0.0009	0.0202 ± 0.0009	0.0202 ± 0.0009	0.0201 ± 0.0008	0.16%
Tissue volume fraction	0.94 ± 0.09	0.97 ± 0.05	0.97 ± 0.05	0.97 ± 0.05	0.97 ± 0.04	0.96 ± 0.05	0.97 ± 0.05	0.96 ± 0.05	1.03%
Intra – neurite Volume fraction	0.44 ± 0.08	0.43 ± 0.07	0.43 ± 0.07	0.42 ± 0.07	0.42 ± 0.07	0.42 ± 0.07	0.42 ± 0.07	0.43 ± 0.07	1.63%
MSE	0.0014 ± 0.0006	0.0014 ± 0.0005	0.0013 ± 0.0005	0.0013 ± 0.0005	0.0013 ± 0.0005	0.0014 ± 0.0005	0.0014 ± 0.0005	0.0014 ± 0.0005	3.55%

Significant values are in bold.

**Fig. 2:**
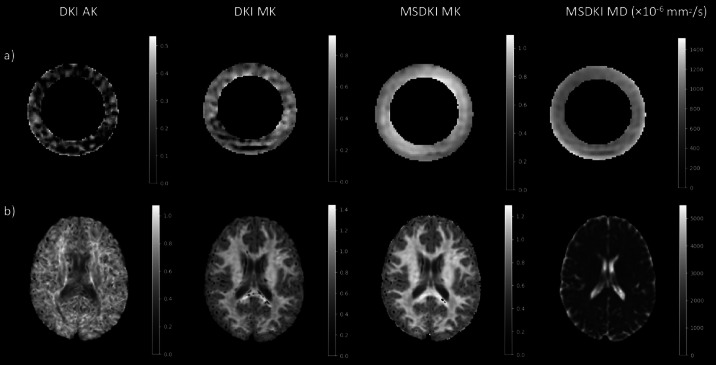
DKI and MSDKI maps obtained in the fiber ring of the phantom (**a**) and in the brain (**b**). From left to right the maps show the DKI AK, DKI MK, MSDKI MK and the MSDKI MD. Except for the MSDKI MD, all the indices are dimensionless and range from 0 to 1.

The DKI and MSDKI metrics are reported in Table 1, in the case of the four scans on different days, and in Table 2, in the case where the phantom was scanned eight times on the same day. The CoVs were lower than the 5% threshold, except for the AK metric. Figure 2 shows the DKI MK assumes unrealistic low values (< 0.3) along the fiber ring, where the fibers are compact and well structured. These low values are not present in the case of the MSDKI MK map (> 0.5), which assumes only axial kurtosis.

**Fig. 3:**
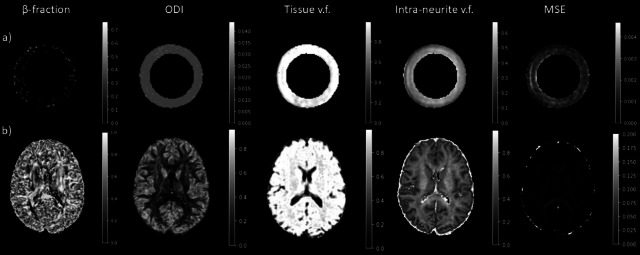
NODDI maps obtained in the fiber ring of the phantom (**a**) and in the brain (**b**). From left to right the maps show the β-fraction, the ODI, the tissue volume fraction (v.f.), the intra-neurite v.f. and the MSE. All the indices are dimensionless and range from 0 to 1.

The results of the Bingham-NODDI fit parameters for the four acquisitions performed in the phantom study are reported in Table 1, along with the CoV corresponding to each metric. The CoVs were lower than the 5% threshold for ODI (1.36%), tissue volume fraction (1.47%) and intra-neurite volume fractions (1.57%). For the β-fraction, the CoV was 6.5%. The diffusion properties of the fiber ring can be seen in Fig. 3, with an ODI close of 0.021 ± 0.002, a tissue volume fraction of 0.98 ± 0.04 and an intra-neurite volume fraction of 0.44 ± 0.08. Table 2 presents the results of the eight consecutive scans. The CoV was below 5% for the β-fraction (2.34%), ODI (0.16%), the tissue volume fraction (1.03%) and the intra-neurite volume fraction (1.63%).

**Fig. 4:**
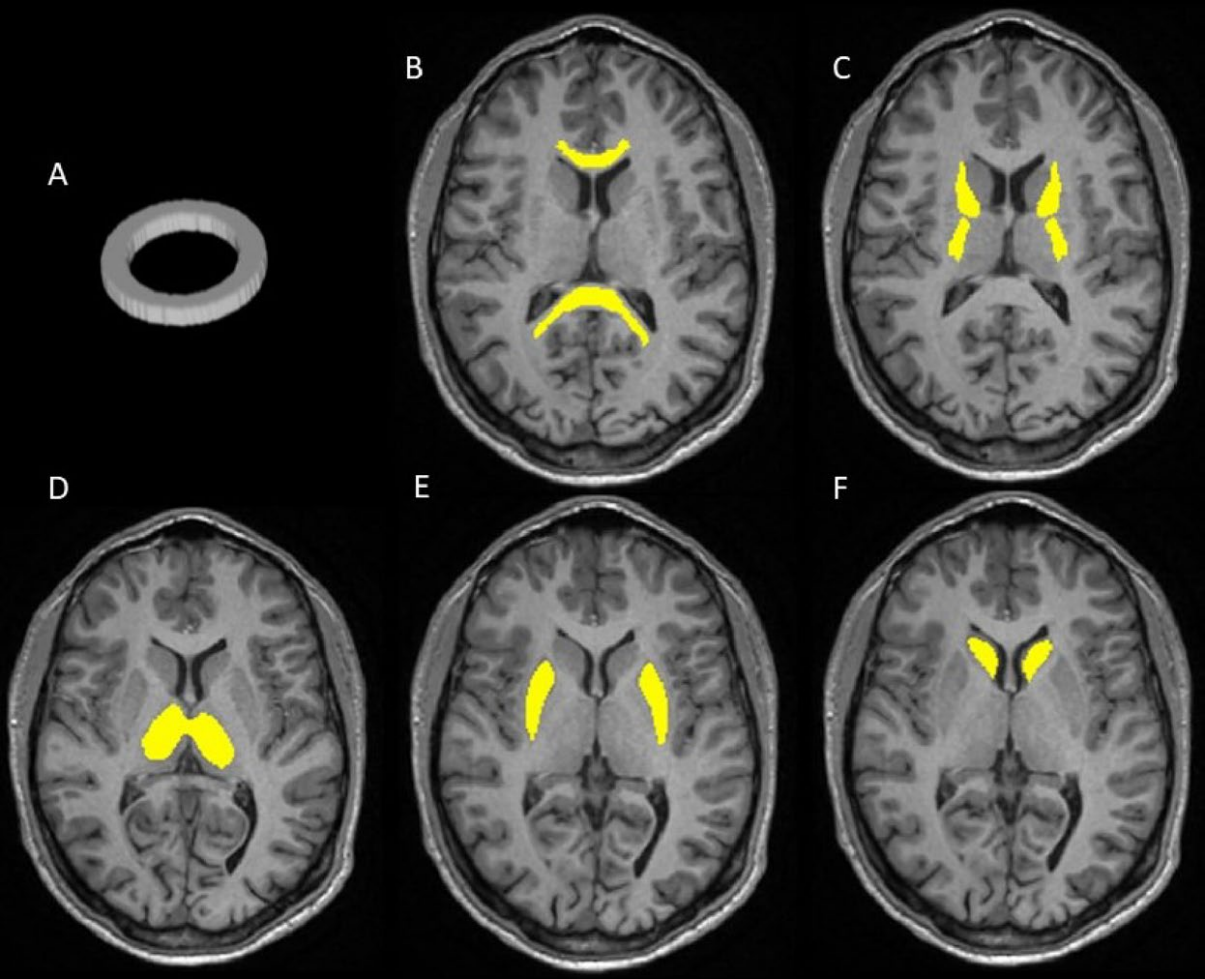
ROIs used to extract the results from the quantitative maps. (**A**) 3D binary mask manually designed to extract the results in the phantom study. ROIs for the in vivo study are the Corpus Callosum (**B**), the Anterior and Posterior limbs of the Internal Capsule (**C**), the Thalamus (**D**), the Putamen (**E**) and the Caudate (**F**). The brain ROIs were defined in the MNI152 space.

In addition to the phantom, a group of four healthy volunteers (HV) was also scanned with the same acquisition protocol. Each HV was scanned twice on the same day, with a 10-minute break between the two acquisitions, with scans occurring over a period of 1 month. Diffusion metrics were extracted from specific ROIs, shown in Fig. 4, and the repeatability of the measurements was assessed with the repeatability coefficient (RC). The DTI and DKI results for the in vivo study are shown in Table 3, which presents the RCs for each metric in each ROI. For all the DTI and DKI metrics, the RCs were low with a maximum value of 0.085 for the MSDKI MK in the Thalamus. The FA and MD maps are shown in Fig. 1, while the DKI maps are shown in Fig. 2. Table 3 also presents the RCs for each NODDI metric in each ROI. The quantitative maps obtained from the analysis are shown in Fig. 3. For each metric, the RC results were low in both white matter and grey matter ROIs, with the highest value of 0.055 for the β-fraction in the Caudate.

**Table 3:** RC results of the in vivo study for the DTI, DKI and NODDI metrics.

	Genu corpus callosum	Splenium corpus callosum	Anterior limb of internal capsule	Posterior limb of internal capsule	Thalamus	Caudate	Putamen
FA	0.022	0.014	0.017	0.027	0.017	0.0068	0.015
MD (×10^− 3^ mm^2^/s)	0.036	0.028	0.034	0.035	0.035	0.030	0.039
DKI MK	0.059	0.14	0.039	0.025	0.065	0.047	0.071
DKI AK	0.018	0.013	0.027	0.021	0.048	0.040	0.034
MSDKI MK	0.039	0.46	0.0051	0.052	0.085	0.084	0.059
MSDKI MD (×10^− 3^ mm^2^/s)	0.019	0.022	0.019	0.022	0.014	0.0091	0.013
β-fraction	0.027	0.0064	0.035	0.021	0.0037	0.055	0.028
ODI	0.0047	0.0073	0.011	0.0035	0.0041	0.0088	0.0087
Tissue volume fraction	0.027	0.029	0.035	0.042	0.0072	0.0091	0.0029
Intra-neurite volume fraction	0.023	0.013	0.024	0.025	0.042	0.024	0.045
MSE	0.00078	0.0011	0.00090	0.0014	0.00042	0.00029	0.00037

## Discussion

A significant challenge in advancing diffusion MRI for microstructural assessment is to overcome the absence of standardized methods for validating the longitudinal stability and reproducibility of derived metrics^35^. This lack of a reference standard limits the reliability of interpretation, particularly when comparing data across time points or different research centers, where technical and physiological factors can introduce variability^33,34^. The purpose of this work was to assess the use of a phantom to aid in the assessment of consistency and repeatability of metrics derived from the DTI, DKI and Bingham-NODDI models over time. The low CoVs, generally below 5% for most metrics across inter-day (Table 1) and consecutive intra-day (Table 2), demonstrated the strong stability and reliability of the phantom. This, in turn, supports the consistency of the results obtained from the DTI, DKI, and Bingham-NODDI models over time.

The first part of the study evaluated the use of the phantom, as a proxy for human brain tissue, through repeated acquisitions of DTI data, with subsequently estimated microstructure model parameters. The low ODI consistently measured around 0.020 ± 0.001, together with the FA of approximately 0.8 ± 0.1 in the phantom, suggest a well-organized structure of fibers within the voxel consistent with the stated manufacturing data. The results of the four measurements performed on different days, leading to a CoV of 1.36%, show a strong reproducibility of the ODI value in the phantom over time. The β-fraction CoV slightly above 5% may indicate a non-uniform distribution of values within the fiber ring. This could be attributed to model fitting instability or structural variability in the phantom, leading to a higher standard deviation in the measurements. The tissue volume fraction estimates the amount of tissue, including cellular structures, that occupies the volume of a voxel. With the lowest value measured being 0.94 ± 0.09, it indicates that the fiber ring can mimic white matter, while excluding the CSF. Moreover, the results are consistent over time, as shown by the CoV 1.47% observed across the four measurements. The intra-neurite volume fraction represents the proportion of the voxel’s volume that is occupied by the fibers of the phantom, excluding the non-tissue compartment. For the intra-neurite volume fraction, the results showed repeatability over time with a CoV of 1.57%.

To determine if repeated data acquisition and heating of the MRI gradient coils affect phantom measurement accuracy, a series of measurements and analyses were conducted. The low CoV, again generally below the 5%, associated with each parameter indicates a high level of stability in the DTI, DKI, and Bingham-NODDI metrics across all eight successive acquisitions. The results confirm the stability of the phantom, and the consistency of the methodology used in this study. This robustness is essential for validating the use of phantoms to ensure measurement accuracy, especially in situations that require multiple consecutive scans or longitudinal studies. The stability is critical because, unlike in vivo measurements which are subject to physiological fluctuations, the phantom provides a controlled environment. This allows for a clear assessment of the stability of the methodology, sequence performance, and diffusion metrics, effectively addressing the need for a ground truth. The consistency observed during repeated scans within a single session, even considering potential factors like gradient heating, reinforces the utility of phantoms for quality assurance protocols.

In the in vivo study, a clear visual distinction was observed between white and grey matter in the ODI maps. This contrast arises from the difference in the ODI index between the two tissue types, with white matter having a significantly lower ODI than grey matter. The lower index value indicates a more structured neurite arrangement in white matter, with very little neurite dispersion. On the other hand, the higher ODI in grey matter suggests greater neurite orientation dispersion. Furthermore, white and grey matter presents with a tissue volume fraction almost equal to one, highlighting the very low presence of inter-cellular water in the healthy brain. It was evident that the intra-neurite volume fraction is greater in white matter than in grey matter, indicating a higher neurite density. The low RCs obtained for the in vivo study highlight the consistency of the NODDI metrics over time, also considering the possible influence of confounding physiological factors that may be encountered in brain studies. Overall, this analysis highlights the inherent stability of DTI, DKI, and NODDI metrics, demonstrating intra-session repeatability within individual healthy participants and robust consistency across separate scanning days for different volunteers. The repeated measurements show minimal fluctuations in all the considered metrics, such as FA, MD, β-fraction, ODI, tissue volume fraction, and intra-neurite volume fraction. As a result, these metrics present a consistent measurement framework, well-suited for situations that demand repeatable results across multiple scans over time.

### Limitations

This was a single site study as the data were acquired with the same scanner. The work should next be extended to a multi-center study, possibly including acquisitions with different magnetic field strengths, to consider the possible variability in the results due to the acquisition of data with different scanner manufacturers.

The sample size of the volunteer cohort was relatively small. Increasing the sample size in future work would increase statistical power and improve the generalizability of the findings. Moreover, examining HV across the lifespan would account for the effect of the age-related physiological changes, thus increasing the robustness of the method.

## Conclusion

This study highlights the potential role of a DTI phantom as a gold standard reference to validate the stability and reliability of scanners and diffusion MRI models over time, an approach rarely addressed in the existing literature, with a focus on the Bingham-NODDI and DKI frameworks.

The phantom scans demonstrated excellent consistency and repeatability, with low coefficient of variation (CoV) across repeated acquisitions, confirming the robustness of the methodology for longitudinal studies. Moreover, the stability of the phantom across repetitive acquisitions, even within potential gradient coil heating effects, further proves its value as a standard for assessing measurement reliability.

In addition to phantom-based validation, in vivo scans of four healthy volunteers showed minimal variability between repeated acquisitions, further supporting the reliability of the NODDI/DKI models in capturing subtle changes in tissue microstructure over time. However, it is the use of the phantom that establishes a standardized, reproducible reference, addressing a significant requirement in diffusion MRI studies by providing a solid foundation for method validation and cross-study comparisons.

The next research phase will involve a multi-center study to investigate inter-scanner variability, potentially incorporating different magnetic field strengths. Integrating a standardized phantom as part of calibration protocols would not only ensure consistent diffusion MRI measurements across different MRI systems but also set a precedent for the field, advancing the reliability and reproducibility of diffusion MRI studies. There are efforts in the field to provide a standardized phantom as part of calibration protocols, which is an encouraging step toward multi-centre trials using advanced diffusion metrics, and a comparison of the available phantoms would be of interest to assess their utility in the clinical setting.

## Methods

### Phantom

**Fig. 5:**
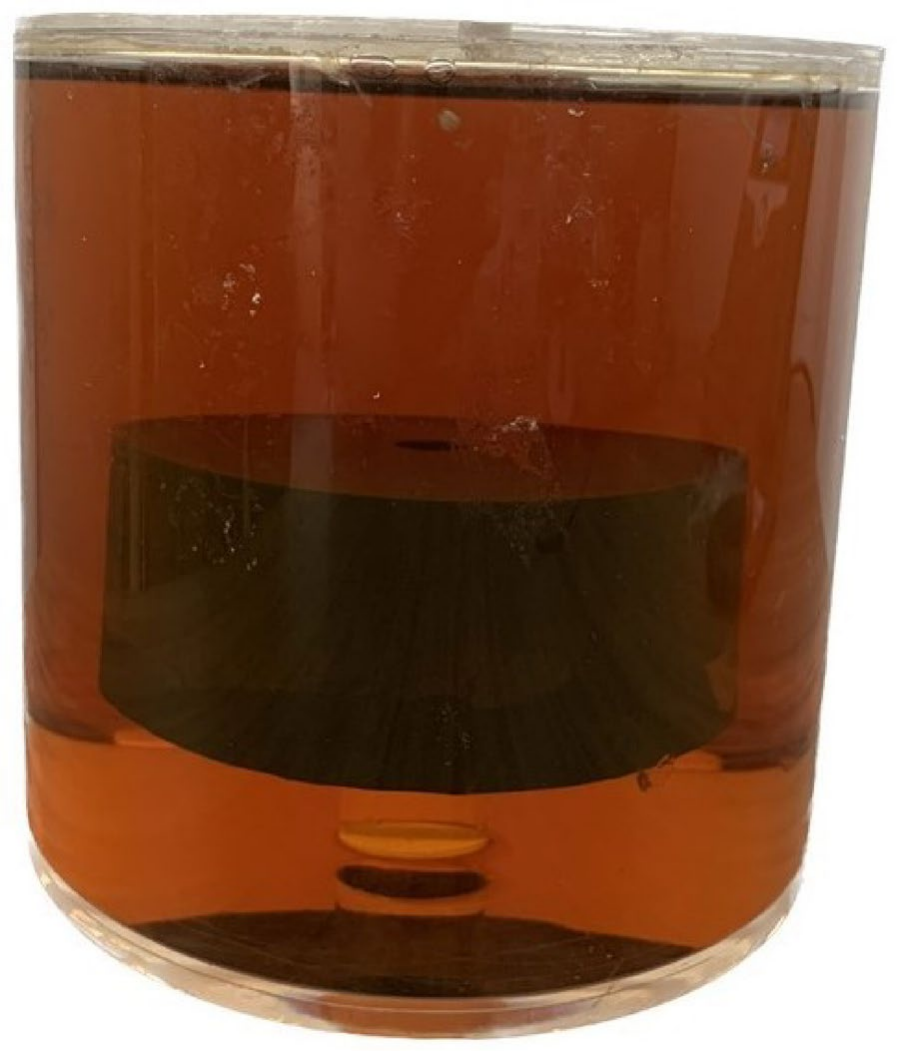
The phantom is composed of a fiber ring with uniform anisotropy at each position, which is embedded in a homogeneous medium of water and sodium chloride to mimic restricted anisotropic diffusion in brain tissue.

The phantom used in the study (German Cancer Research Center (DKFZ), Heidelberg, Germany)^36^ (Fig. 5) is composed of a fiber ring with uniform anisotropy at each position, which is embedded in a homogeneous medium of distilled water and sodium chloride, with a concentration of 83 g of NaCl per kilogram of water. This fluid constitution enables an orientation-independent and reliable use of the phantom for evaluation purposes. The fibers are made of a synthetic, extremely fine polyester fiberfill of diameter 15 μm and are winded around an acrylic plastic spindle. The polyfill is made of a filament yard which consists of continuous long strands of polyester fibers, with a linear mass density of 50 decitex. The outer fiber strand has a diameter of 60 mm and a thickness of 10 mm. Water is present between the fibers to simulate restricted anisotropic diffusion in white matter. The polyamide fibers winded around the plastic spindle are contained inside a cylindrical phantom container of diameter 150 mm and height 150 mm. As per the information provided by the manufacturer, the fractional anisotropy of the phantom is 0.78 ± 0.02^36^.

### Participant recruitment

All experiments were performed in accordance with the relevant guidelines and regulations. Written informed consent was obtained from all subjects prior to their inclusion in the study. Data were collected under an ethically approved protocol ‘Metabolic Imaging of Neurological Disease (MIND)’ Ethics Ref: 20/SC/0441. Four Healthy Volunteers (HV) with no prior history of neurological or psychiatric conditions were recruited for this study, 29 ± 3 years (mean age ± std), one female.

### Data acquisition

The phantom and participants were scanned using a 3T scanner (General Electric Healthcare (GE), Waukesha, WI, USA) with a 21-channel head and neck coil.

For each scan, the phantom was carefully placed inside the head coil in such a way that the axis of the container was aligned with the $\:{B}_{0}$ field of the scanner. The fiber ring of the phantom was placed at the isocenter of the MRI scanner. The DTI protocol for the phantom consisted of a single-shot, spin echo-based, and diffusion-weighted echo planar imaging sequence. Repetition time (TR) = 6000 ms, echo time (TE) = 70.1 ms. Diffusion data was acquired with a multi-shell protocol, along 90 distinct diffusion directions with two corresponding b-values: 30 directions with a b-value = 1000 s/mm^2^ and 60 directions with a b-value = 2600 s/mm^2^, 9 b = 0 s/mm^2^ images randomly dispersed in between the diffusion-weighted images, ASSET (Array Spatial Sensitivity Encoding Technique) = 2 was used, field of view (FOV) = 240 mm^2^acquisition matrix = 96 × 96, slice thickness = 2.5 mm, number of slices = 32, receiver bandwidth = 250 kHz. The sequence was 12 min long and was adapted to output a b = 0 reversed polarity phase encoded acquisition in the same acquisition. Phantom data sets were acquired as the first scans of the day 4 times over a 2-month period. A further 8 data sets were acquired in one day with no disruption between scans.

The imaging protocol for HV included a T_1_-weighted and diffusion-weighted scan. Whole-brain T_1_ weighted 3D volumes were acquired with a Magnetization Prepared RApid Gradient Echo sequence (MPRAGE) with the following parameters: TR = 2584 ms, Inversion Time (TI) = 1058 ms, TE = 2.9 ms, 80% Phase FOV, Acquired Voxel Volume = 1 mm^3^Flip angle = 8°, FOV = 256 mm. For the diffusion-weighted scan, the acquisition parameters were the same as the phantom, except for the use of simultaneous multi-slice (multiband) acceleration factor of 2. DTI slices were aligned to the corpus callosum. After a subject was scanned with all MR sequences in a scan session, the subject left the scanner table, walked around the MRI controlled area for 10 min, and was repositioned for the second scan session, where the same sequences used in the first session are repeated. The four healthy volunteers were scanned over a period of one month.

### Post-processing

DTI data were processed using FSL^38,39^ using the *topup*^40^ and *eddy*^41^ to correct for the distortions due to the local inhomogeneities of the magnetic field and the presence of eddy currents. The implementation of the Bingham-NODDI model was performed by using the Diffusion Microstructure Imaging in Python (Dmipy) framework^42^. Dmipy is a Python-based toolkit that allows flexible and efficient implementation of diffusion MRI models and parameter estimation. The exact code used for fitting the diffusion data to the Bingham NODDI model can be found on the Dmipy GitHub page^43^. DKI and MSDKI models fit using the Diffusion Imaging in Python (DIPY) package^44^.

A region of interest (ROI) was manually drawn in the ring region of the DTI phantom, shown in Fig. 4. Phantom data was processed to correct for distortion artefacts and then voxels within the fiber ring were fitted to the DTI, DKI and Bingham-NODDI models. Mean and standard deviation of each metric were extracted from the ring ROI.

Brain data were corrected for distortion artefacts as above and T_1_ volumes were registered to the MNI space using the *flirt*^45–47^ and *fnirt*^48^ functions of FSL^38,39^. The DTI, DKI and Bingham-NODDI models were fit to the corrected diffusion data and the obtained maps were registered to the MNI space. Mean and standard deviation of each metric were extracted from specific ROIs, shown in Fig. 4, defined in the MNI space: Genu and Splenium of the Corpus Callosum, Anterior and Posterior limbs of the Internal Capsule for white matter, and Caudate, Thalamus and Putamen for grey matter. These ROIs were obtained from the Harvard-Oxford FSL atlas^49^ and an erosion of the mask was performed to avoid possible partial volume effects.

### Statistical analysis

In the case of the phantom study, the coefficient of variation (CoV) was calculated to assess the stability and consistency of the results over time. A 5% threshold was used to determine the consistency of the results over time.

For the in vivo study, the repeatability coefficient (RC) defined as

$$\:RC=1.96\cdot\:\sqrt{\frac{{\Sigma\:}{\left({m}_{2}-{m}_{1}\right)}^{2}}{n}}$$.

was computed to assess the maximum difference that one would expect to observe between two measurements $\:{m}_{1}\text{\:and\:}{m}_{2}$ taken on the same subject under the same conditions, computed over all the $\:n$ subjects in the dataset. A smaller RC value signifies lower measurement variability and, consequently, higher consistency between the two measurement techniques.

## Data Availability

Data are available from the corresponding author upon reasonable request.
